# Supplementary material for: Peptide Specific Nanoplastic Detection Based on Sandwich Typed Localized Surface Plasmon Resonance
Source: Nanomaterials (Basel). 2021 Oct 28;11(11):2887. doi: 10.3390/nano11112887 (PMC8617854; doi:10.3390/nano11112887)
Supplement: Supplementary file 1 [file nanomaterials-11-02887-s001.zip › nanomaterials-1399201-supplementary.pdf]

Supplementary Materials

Peptide Specific Nanoplastic Detection Based on Sandwich Typed Localized Surface Plasmon Resonance

Seungju Oh [1], Hyeyeon Hur [1], Yoonjae Kim [1], Seongcheol Shin [1], Hyunjeong Woo [2], Jonghoon Choi [2,*] and Hyun Ho Lee [1,*]

[1] Department of Chemical Engineering, Myongji University, Yongin-Si 17058, Korea; sjtleo55@gmail.com (S.O.); hhuh9801@gmail.com (H.H.); yse459@gmail.com (Y.K.); lms4278@gmail.com (S.S.)

[2] Department of Biomedical Engineering, School of Integrative Engineering, Chung-Ang University, Seoul 06974, Korea; hyunjeong1226@gmail.com

* Correspondence: nanomed@cau.ac.kr (J.C.); hyunho@mju.ac.kr (H.H.L.)

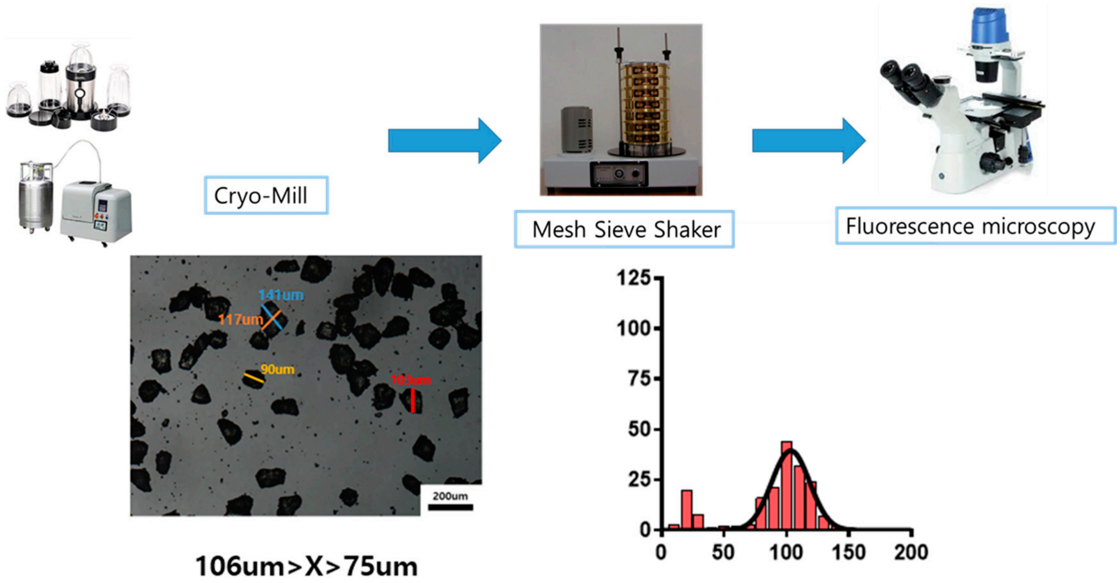

Figure S1. Procedure and photograph of cryo-grinded PS.

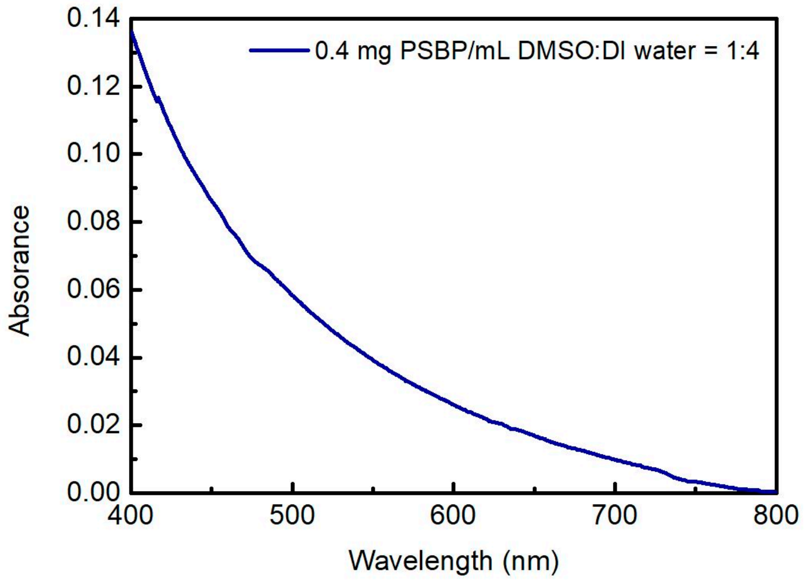

Figure S2. UV-Vis measurement results obtained via PSBP in DMSO.

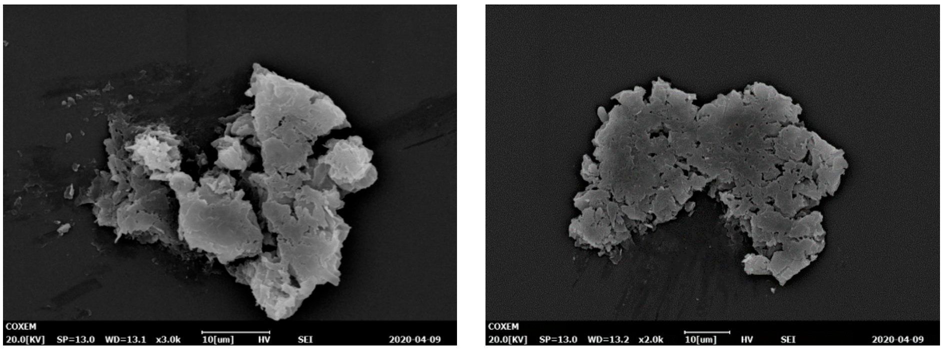

(**a**)

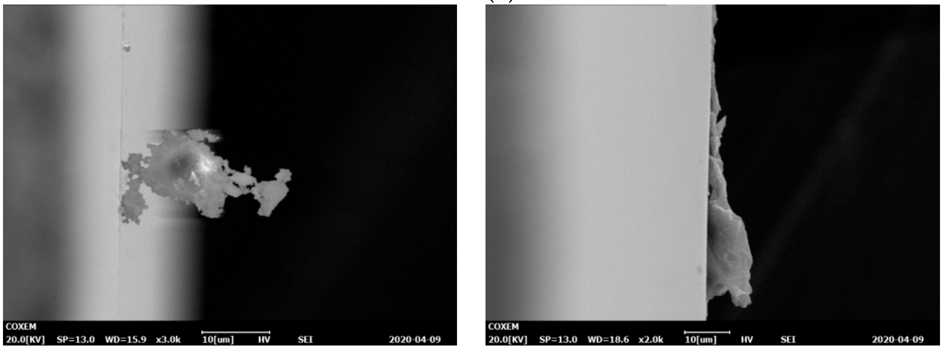

(**b**)

Figure S3. SEM measurement images for 1 mg/mL PS sample binding (**a**) the top of the LSPR chip, and (**b**) the side of the LSPR chip.

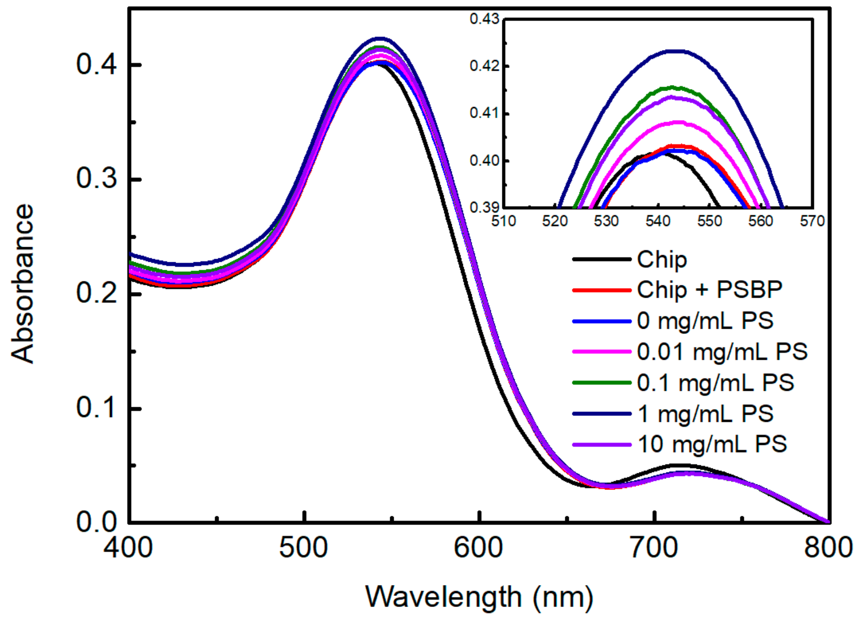

(**a**)

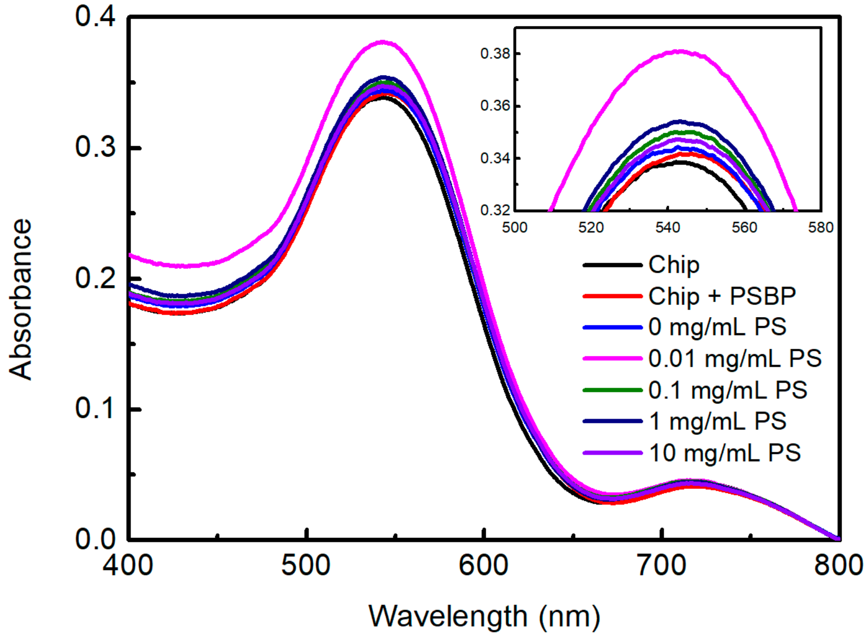

(**b**)

Figure S4. UV-Vis measurement results obtained via (**a**) Non-Sandwich assay and (**b**) Sandwich assay of 75~106 μm PS fragments.

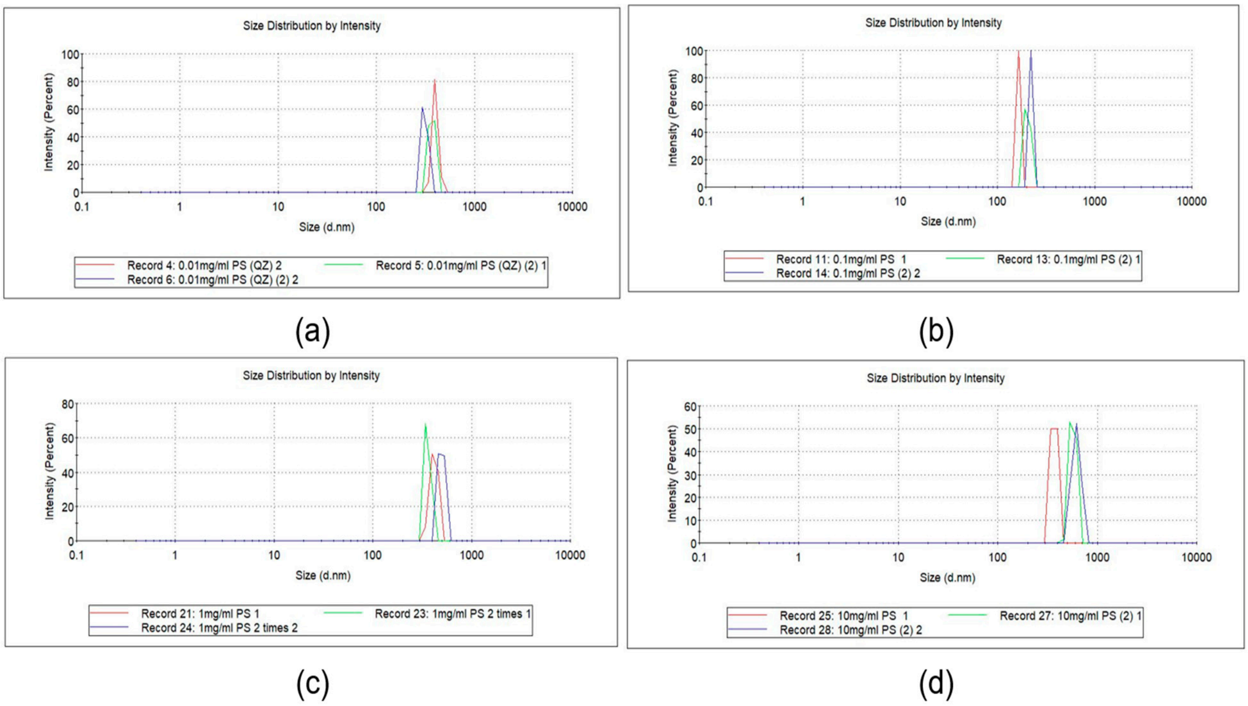

Figure S5. Sizes of particles in the (**a**) 0.01 mg/mL solution, (**b**) 0.1 mg/mL solution, (**c**) 1 mg/L solution, and (**d**) 10 mg/mL solution, as measured using dynamic light scattering (DLS).

Figure S5 shows the dynamic light scattering (DLS) results for each PS concentration solution, which shows the number of nanoparticles in the solution according to size.

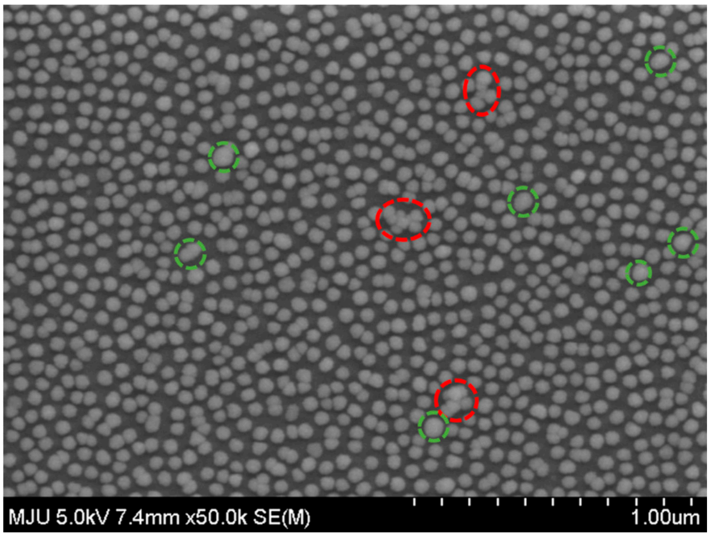

Figure S6. FE-SEM measurement results for the sandwich assay of 75~106 µm PS fragments.

In Figure S6, the sandwich-bound sites as example are denoted by red circles, whereas the non-sandwiched sites associated with 5 nm Au NPs are denoted by green circles. It can highlight larger sized individual Au NPs with circles than unbound probe Au NPs after solely binding of nanoplastic-conjugated 5 nm Au NPs.

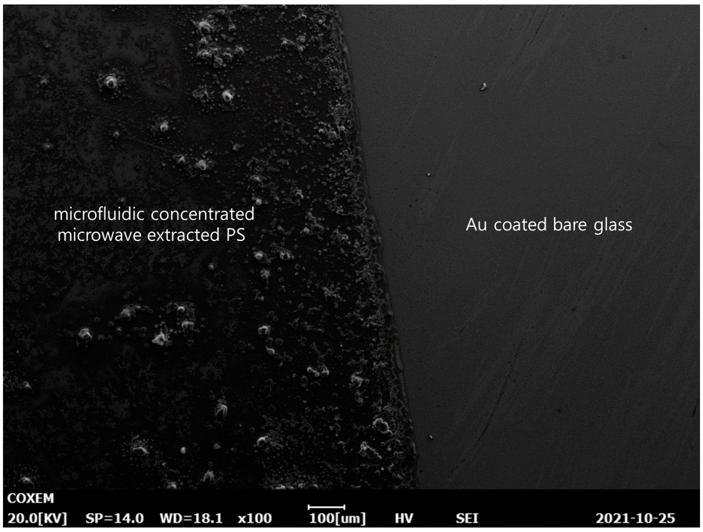

Figure S7. SEM image of microfluidic concentrated microwave-boiled styrofoam particles.
